# Effect of Implementation of HEART Chest Pain Protocol on Emergency Department Disposition, Testing and Cost

**DOI:** 10.5811/westjem.2020.9.48903

**Published:** 2021-02-04

**Authors:** William E. Bylund, Peter M. Cole, Michael L. Lloyd, Anastasia A. Mercer, Amanda K. Osit, Sarah W. Hussain, Matthew W. Lawrence, Micah J. Gaspary

**Affiliations:** Naval Medical Center Portsmouth, Department of Emergency Department, Portsmouth, Virginia

## Abstract

**Background:**

Symptoms concerning for acute coronary syndromes (ACS) such as chest pain and dyspnea are some of the most common reasons for presenting to an emergency department (ED). The HEART score (history, electrocardiogram, age, risk factors and troponin) was developed and has been externally validated in an emergency setting to determine which patients with chest pain are at increased risk for poor outcomes. Our hospital adopted a HEART score-based protocol in late 2015 to facilitate the management and disposition of these patients. In this study we aimed to analyze the effects of the adoption of this protocol. Prior studies have included only patients with chest pain. We included both patients with chest pain and patients with only atypical symptoms.

**Methods:**

This was a retrospective chart review of two cohorts. We identified ED charts from six-month periods prior to and after adoption of our HEART score-based protocol. Patients in whom an electrocardiogram and troponin were ordered were eligible for inclusion. We analyzed data for patients with typical symptoms (chest pain) and atypical symptoms both together and separately.

**Results:**

We identified 1546 charts in the pre-adoption cohort and 1623 in the post-adoption cohort that met criteria. We analyzed the first 900 charts in each group. Discharges from the ED increased (odds ratio [OR[1.56, P<.001), and admissions for cardiac workup decreased (OR 0.46, P <.001). ED length of stay was 17 minutes shorter (P = .01). Stress testing decreased (OR 0.47, P<.001). We estimate a cost savings for our hospital system of over $4.5 million annually. There was no significant difference in inpatient length of stay or catheterization rate. When analyzing typical and atypical patients separately, these results held true.

**Conclusion:**

After adoption of a HEART score-based protocol, discharges from the ED increased with a corresponding decrease in admissions for cardiac evaluations as well as cost. These effects were similar in patients presenting without chest pain but with presentations concerning for ACS.

## INTRODUCTION

Acute coronary syndromes (ACS) include myocardial infarction and unstable angina. The most common symptoms in ACS include chest pain, dyspnea, fatigue, and weakness.^1^ These symptoms are common reasons for presentation to emergency departments (ED). Chest pain itself accounts for approximately 8–10% of ED visits each year nationwide for adults aged 15 years and older.^2^ Ruling out acute myocardial infarction is generally straightforward, but subsequently identifying which patients are at risk for having a major cardiac event in the near future and arranging appropriate access to further screening can be costly, challenging, and risk prone.^3^,^4^ For chest pain patients, the American Heart Association’s 2010 guidelines recommend stress tests to be completed in the first 72 hours of the patient’s visit. No formal guidance exists for patients with atypical symptoms.^5^ This requirement has led to a high number of inpatient stays for cardiac observation and risk-stratification testing with an associated financial burden.^4^

In a study by DeVon et. al, 65–74% of patients with ACS reported chest pain; of those, only 43–53% reported chest pain or discomfort as a chief complaint.^1^ The HEART score was developed for use in an emergency setting for patients presenting with chest pain. The score has been prospectively and externally validated and is widely used to aid in risk stratification and to safely reduce unnecessary inpatient resource utilization.^6^–^8^ A score is calculated from its component elements: history, electrocardiogram [ECG], age, risk factors, and troponin.^9^

**Figure 1:**
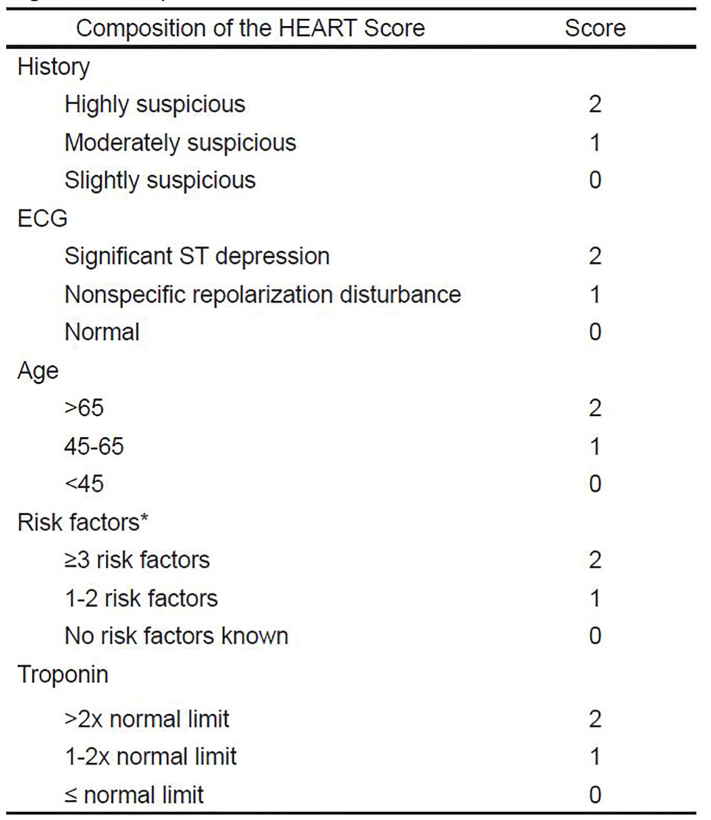
Composition of the HEART score.

The HEART score attempts to distinguish low-risk patients who can be safely discharged from the ED, from patients at a higher risk for a major cardiac event (MACE) defined as death, non-fatal myocardial infarction (MI), or revascularization procedure within a six-week period. The HEART score has been shown to be equal or superior to other scoring systems such as TIMI or GRACE.^10^ The HEART score consists of five factors, each assigned a score of 0, 1, or 2 points; the sum of all five comprises the HEART risk score for potential ACS patients (Figure 1).^6^,^9^

A HEART score of 0–3 is considered low risk and corresponds to a less than 2% risk of MACE within six weeks and supports discharge from the ED without further workup or evaluation; a score of 4–6 is medium risk, corresponding to a 5–20% risk of six-week MACE.^6^ Patients with a medium risk HEART score warrant cardiology evaluation for admission for clinical observation and further cardiac workup. A score of ≥ 7 is considered high risk, conveying a 50–72% risk of six-week MACE and supports initiation of invasive treatment with minimal delay.^6^,^11^ In a retrospective, multicenter analysis, patients with HEART scores 0–3 had a 0.99% rate (3/303 cases) of MACE within six weeks of presentation; those with scores 4–6 and 7–10 had rates of 11.6% (48/413) and 65.2% (107/164), respectively.^6^ It has been further proposed that using HEART scores in combination with zero and three-hour serial troponin measurements reduced hospital length of stay, increased early discharges, and decreased objective cardiac testing.^7^,^12^ This data would indicate that the HEART score is a reliable noninvasive predictor of outcome in this treatment population and can be a valuable tool for safe and efficient patient management in the EDs.

**Population Health Research Capsule**

What do we already know about this issue?

Use of the patient’s history, electrocardiogram [ECG], age, risk factors, and troponin (HEART) score to help increase discharges and reduce downstream testing has been externally validated in emergency department (ED) patients with chest pain.

What was the research question?

Can a HEART-based protocol improve care in patients with both typical and atypical signs of acute coronary syndrome (ACS)?

What was the major finding of the study?

Our HEART-based protocol increased ED discharge rates even in patients with only atypical signs of ACS.

How does this improve population health?

A HEART score-based pathway has the potential to safely increase ED discharge and reduce downstream testing even among patients with only atypical signs of ACS.

The Naval Medical Center Portsmouth (NMCP) evaluates over 65,000 patients annually in its 54-bed emergency department (ED). The patient population includes active duty military members, their families, some retirees, and veterans. Generally speaking, the population is younger and healthier than that of the average community ED. In the fall of 2015, the NMCP ED instituted a protocol based on the HEART score. For patients presenting with symptoms believed to be related to possible ACS, an ECG and troponin are performed. STEMI patients are immediately prepared and sent for percutaneous coronary intervention in our catheterization laboratory. NSTEMI patients are admitted for observation and treatment by our cardiology team. The remainder are entered into the HEART score-based protocol.

In accordance with other studies,^6^,^7^,^11^,^12^ we slightly modified the original HEART pathway to add a second troponin test three hours after the first for patients who present with less than six hours of chest pain. Patients with HEART scores of three or less are discharged from the ED with primary care or cardiology follow-up within 72 hours. Patients with HEART scores of 4–6 are evaluated by the cardiology team for inpatient admission and evaluation or are placed in an observation status in the ED for risk stratification. Patients with HEART scores greater than 6 are generally admitted for treatment and evaluation.

The purpose of this study was to examine how the implementation of our new institutional HEART score-based protocol affects ED disposition (admission vs discharge). Secondarily, we evaluated ED length of stay (LOS), number of stress tests completed, cardiac catheterization rates, and rates of MACE before and after implementation of the protocol. Our protocol does not specifically address patients with purely atypical symptoms. However, ACS is often a concern and a score is easily calculated for these patients. For analysis, we included all patients in whom a troponin and ECG were ordered by the ED team regardless of their chief complaint or reported symptoms.

## METHODS

This was a retrospective chart review study comparing two six-month periods, one prior to implementation and one after adoption of the HEART score-based chest pain protocol. We used procedures outlined in Kaji’s paper on retrospective reviews in the ED as a guide for design and data abstraction.^13^

### Chart Review

We screened medical records using an electronic health record (EHR) (T-system EV, Plano, TX). Records were collected for all patients between the ages of 30–89 who had a troponin test and an ECG ordered in the ED during a six-month period prior to implementation of the chest pain protocol (January 1, 2015–June 30, 2015) and a corresponding annual period after implementation (January 1, 2016–June 30, 2016). The post-implementation period started five months after adoption of the protocol to ensure a washout and standardization period. During both periods, we used two types of non-high sensitivity troponin tests. One is a point of care test (i-STAT cardiac troponin I, Abbott Diagnostics, Chicago, IL), and the other a standard lab assay (Vitros 5600 Troponin I, Ortho-Clinical Diagnostics, Raritan, NJ); the two tests were considered equal for the purposes of the study using their individual reference ranges (normal values for i-STAT < 0.02, and less than 0.034 for laboratory assay).

Using the troponin lab and ECG order as a triggering event in the screening, we identified 1546 records in the pre-implementation group and 1623 in the post-implementation group. The first 900 charts in each cohort were used for analysis. For each group, trained data abstractors manually abstracted required data elements from the EHR and entered this information in a password-protected spreadsheet (Microsoft Excel, Redmond WA). Data was abstracted from the ED EHR), the outpatient EHR (AHLTA, Unissant Inc, Herndon, VA) and the inpatient EHR (Essentris, CliniComp, Intl, San Diego, CA) to complete the password-protected dataset. Data abstractors were not blinded to study objectives. Patient names were de-identified with a separately held subject ID key. Patients who were diagnosed with ST-elevation myocardial infarction (STEMI) or non-ST elevation myocardial infarction (NSTEMI) were excluded, as were those whose troponin results and ECGs were missing from the EHR system (Figure 1). The included records were evaluated by a physician who used a modified spreadsheet containing a randomized listing of the patient’s chief complaint and history of present illness (HPI) to calculate a score for the history portion of the HEART score. This physician was blinded to the patient’s group (pre or post). We used the original and validation studies of the HEART score as a guide for scoring the history.^6^,^11^ If the chief complaint or the HPI included chest pain, pressure or discomfort, the patient was included in the typical group. Otherwise, the patient was placed in the atypical group for analysis.

ECG interpretations, age, risk factors, and troponin were taken directly from the chart. If the ECG interpretation was not available in the chart, the actual ECG was evaluated and scored by a physician blinded to the patient’s cohort and medical record according to the HEART algorithm. Zero points were assigned to ECGs that were normal, one point if there were non-specific repolarization disturbances.

### Outcomes

The primary outcome was patient disposition (admission vs discharge). For the purpose of this study, “admission” was defined as a transfer of care to ED observation, inpatient internal medicine, or the cardiology service. Secondary outcomes included ED LOS, number of stress tests performed, number of catheterizations performed (and the results), and rate of MACE. We indirectly estimated cost savings by using standard costs obtained by our business affairs department for cardiac admissions, floor admissions, cardiology outpatient follow-up, stress testing, and catheterizations.

### Analysis

We assumed alpha 0.05 and beta 0.2 for our sample size estimate. To determine a 10% difference in ED discharge rates (two-sided), 380 subjects per group were required. To determine a 5% difference (two-sided), 1320 subjects per group were required. We performed interim power analysis after 900 records had been collected for each side, and the numbers collected were deemed sufficient.

Baseline patient characteristics (history, age, EKG, risk factors, troponin category) were converted into categorical data based on the HEART score (Figure 1). Results were compared before and after implementation of the HEART-based chest pain protocol. For categorical data, differences between the groups were evaluated using chi-square and Fisher’s exact tests. We evaluated continuous data using two-sided Student’s T tests. We performed logistic regression analysis to control for potential confounders including differences in HEART scores between the pre- and post-protocol groups.

We examined the reason for visit, which was recorded by the front desk staff, the chief complaints entered by the nurse and physician, and the HPI sections in the EHR. The HPI section also included a basic review of systems. If there was any mention of chest pain, pressure or discomfort (eg, chest + “pain,” “discomfort,” “pressure,” “squeezing,” or “heaviness”) the patient was placed in the typical category. Otherwise, the patient was placed in the atypical category.

## RESULTS

### Chart Review

**Figure 2:**
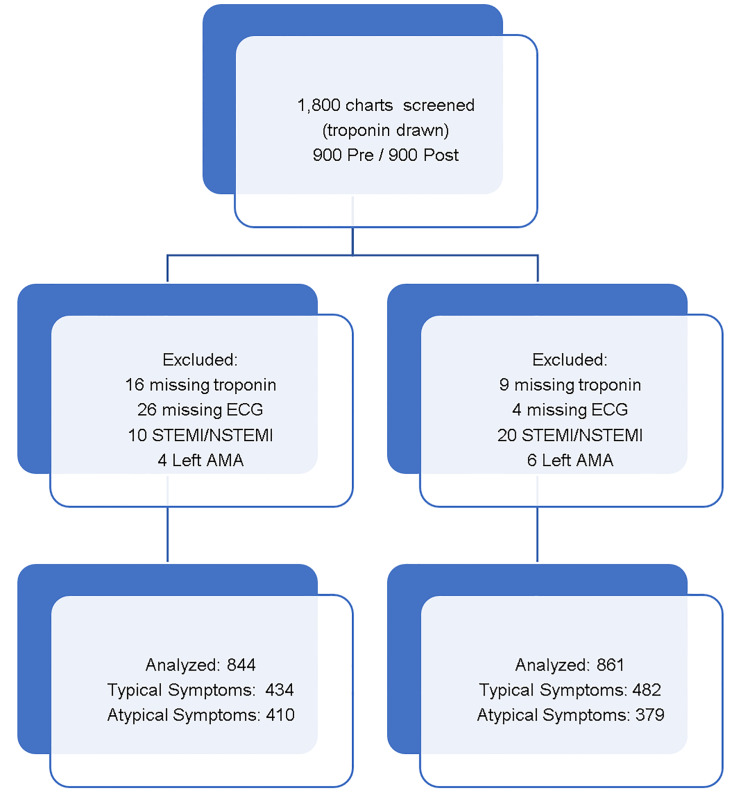
Inclusion criteria flow chart. *ECG*, electrocardiogram; *STEMI*, ST-segment elevation myocardial infarction; *NSTEMI*, non-ST-segment elevation myocardial infarction; *AMA*, against medical advice.

We analyzed 900 records in the pre-implementation group and 900 seasonally matched records in the post-implementation group. To directly compare our study to similar studies, we grouped our records into two main categories. Patients with typical symptoms and atypical symptoms were analyzed together and separately. Pre-protocol, we excluded two patients with STEMI and eight patients with NSTEMI. We also excluded 16 patients for missing troponins, 26 patients for missing ECGs, and four patients who left against medical advice (AMA). Post-protocol, we excluded seven patients with STEMI and 13 patients with NSTEMI. We also excluded nine patients for missing troponins, four patients for missing ECGs and six patients who left AMA. This left 844 patients in the pre-protocol cohort, 434 of whom demonstrated typical ACS symptoms and 410 with atypical symptoms. In the post-protocol cohort, we included 861 records, 482 of which demonstrated typical symptoms and 379 with atypical symptoms (Figure 2).

Patients in the pre-protocol cohort were more likely to have normal ECGs, were older, had more risk factors, and were more likely to have a positive troponin. When combined into a total HEART score, there were more low-risk patients in the post cohort but this did not reach statistical significance (*p* = .06). We adjusted for these differences using logistic regression to account for the HEART score category, which takes the differences seen in age, troponin, and risk factors into account. The regression dampened some findings but did not significantly change results in any outcome and are included in the respective outcome sections.

Typical vs atypical symptoms: About half of patients in each cohort presented with chest pain as a chief complaint (51%, 56%). Within our exclusions for NSTEMI, 7/21 presented with atypical symptoms (33%). For STEMI patients who were excluded, 1/9 (11%) presented with atypical symptoms (dyspnea). Atypical patients were more likely to be scored as 0 for the history portion of the HEART score. This was consistent between cohorts (39% typical vs 86% atypical pre; 39% typical vs 88% atypical post) (Table 1).

#### Primary Outcomes: Patient Disposition

ED discharge rates: For all patients, the discharge rate from the ED increased by 10.8% absolute (odds ratio [OR] 1.56, 1.49 adjusted, *p*<.0001). This trend held true whether the patient had typical chest pain or atypical symptoms (Table 2).

Hospital admission rates: Admissions to the cardiology service decreased by 11.6% absolute, (OR 0.46, 0.45, *p*<.0001). Admissions to other services increased by 1.5% absolute (OR 1.10, 1.15, *p* = .5). This represents a trend but did not reach statistical significance. When analyzed by symptoms, the trend seemed more profound for patients with typical symptoms (OR 1.52) vs atypical symptoms (OR 1.13), but again these differences did not reach statistical significance (*p*= .1 typical, *p*= .5 atypical) (Table 2).

### Secondary Outcomes: Length of stay, stress tests, catheterizations, MACE, and cost

ED LOS: We analyzed two different ED LOS categories for discharged patients. Overall LOS included time in the waiting room. Room to discharge time eliminated the time in the waiting room from analysis. For all discharged patients, overall LOS was 13 minutes shorter in the post-protocol group but this did not meet statistical significance (*p*= .07). Room to discharge time for discharged patients was 17 minutes shorter for all patients (*p*= .012). For typical chest pain patients, room to discharge time was 19 minutes shorter (*p*= .037). For patients with atypical symptoms, ED LOS was 13 minutes shorter but was not statistically significant (*p*= .17) (Table 3).

Inpatient LOS: We analyzed inpatient LOS for all admitted patients. We grouped all admits together to include those admitted to ED observation, those admitted to the cardiology service, and those admitted to other services. If a patient was admitted and discharged on the same day, we considered them admitted for one day. Otherwise we calculated the number of days between admission and discharge. There was a small but significant decrease in inpatient LOS from pre to post (2.62 days to 2.17 days, *p*= .02). Admissions for atypical symptoms were about a day longer than for typical symptoms in both groups (Table 3).

Number of stress tests performed: The number of stress tests performed (which include treadmill/exercise stress tests, stress echocardiograms, and chemical stress tests) decreased by half from pre-protocol to post-protocol (16% to 8%, OR 0.47, *p*<.001). This trend held true when analyzed by symptoms. For typical symptoms, stress tests decreased by 13% absolute (26% to 13%, OR 0.43, *p*<.001). For atypical patients, stress tests decreased by 4% absolute but numbers were low overall (6% to 2%, or 0.36, *p*= .02). Across all groups, the percentage of positive stress tests did not increase or decrease significantly (14% vs 15%, OR 0.99, *p*= 1.0) (Table 4).

Cardiac catheterization rates and results: The number of cardiac catheterizations performed was low for both cohorts and did not change significantly from pre to post (4% vs 3%). Of the catheterizations performed, a greater portion were positive post-protocol (53% vs 77%), but this did not reach statistical significance (Table 4).

MACE: Among the 1705 patients included in the final analysis, six-week follow-up data could not be confirmed for 8% (134 patients). Loss to follow-up was consistent in both groups (7.7% pre, 8.0% post). Follow-up was done by looking through outpatient records for repeat visits more than six weeks after the index visit. Patients with primary care or cardiology care outside of our facility (which is not uncommon) would not be expected to be found in this way. This limited our ability to draw significant conclusions regarding MACE. Among the 92% of patients for whom follow-up was available, there were no missed MACE cases. There were four deaths within the six-week period, but all were admitted to the hospital at the index visit.

Healthcare costs: To calculate savings or cost of the protocol to the hospital, we requested cost information from the hospital business office. We were provided with a list of average costs for various services. In the pre-protocol cohort, the first 900 patients presented over 109 days, and over 98 days for the post-implementation cohort. We calculated the number of events per day over these periods and calculated an annual cost based on these numbers. This method accounts for an increase in visits in the post-protocol period. For cardiology visits, we assumed all extra discharges had a visit with a cardiologist and therefore that the number of outpatient cardiology visits increased. This is likely a significant overestimation, as many low-risk patients follow up only with their primary care provider. This method appropriately biases against our intervention. Cost information is presented in Table 5.

## DISCUSSION

Our study is similar to a study by Hyams in 2018, which showed a similar resource-utilization benefit to a HEART-based protocol. Hyams’ and all other HEART studies to date have included only patients presenting with chest pain.^8^ In this study, by including all patients where an ECG and troponin were ordered, we included and analyzed data from patients presenting with only atypical symptoms of ACS. As far as we know, this is the first study to look at this population. ACS is considered a “can’t miss” diagnosis in the ED. Chest pain is the most common symptom of ACS but is by no means universal.^1^ Ruling out ACS in patients with only atypical symptoms is challenging. In our study, 44–49% of ECGs and troponins were ordered on patients without chest pain, pressure, or discomfort as a chief complaint or anywhere in the history of present illness. This demonstrates a real-world ED approach to evaluating for cardiac ischemia. In our population, 33% of NSTEMI diagnoses resulted from investigation of atypical symptoms. Nine of the 37 (24%) abnormal catheterizations occurred in the subgroup with only atypical ACS symptoms.

Our HEART protocol simplifies ED evaluation and decreases unnecessary hospital admissions for low-risk patients. The protocol enables more rapid disposition and decreased resource utilization for those in whom MI is ruled out. The discharge rate for chest pain improved by 10.8% absolute (48% relative). Our study corroborates prior studies, demonstrating an OR of 0.46 for admission (vs 0.48 in the Hyams study).^8^ Our facility serves primarily active duty military and their families with a smaller portion of retirees. As expected, the percentage of patients with low HEART scores was higher in our population (63–69%) than in other studies (31%, Mahler 2018).^14^ Additionally, our medical system differs significantly from a civilian setting with increased access to care and significantly reduced patient-borne costs that may lower patient threshold to present for care.

ED room to disposition times were 17 minutes shorter after implementation of the protocol. Over a year and over 1500 visits this added up to a significant time savings and improvement in patient flow. Given the frequency of cardiac evaluations in any ED, higher discharge rates and shorter stays help reduce waiting room delays and improve patient access to care. Inpatient LOS decreased slightly as well (0.45 days), but these data are a bit less reliable given that we were only able to consider full days and not portions of days in the analysis. In 2011 Mahler et al suggested that the HEART score could reduce stress testing and cardiac imaging.^15^ Our study shows a similar significant reduction in stress testing. Interestingly, there was a lower proportion of abnormal stress tests in the atypical population in the post cohort (13% vs 30%) although numbers were quite low and differences were not statistically significant. Considering the inherent imperfections in stress testing this may not be an indication of the HEART protocol missing cases. Cardiac catheterization procedures in the pre and post cohorts were also low and not statistically significant but a higher positive catheterization rate (77% vs 53%) in the post cohort may indicate better patient selection.

Although our cost data is indirect and incomplete, based on saving admissions to the cardiac care unit, increasing ED discharges and decreasing admissions and stress tests resulted in an estimated cost savings to our military medical center of approximately $4.5 million annually.

## LIMITATIONS

There were several limitations to our study, with the primary being the retrospective chart review design conducted over a two-year timespan. Other confounding variables may exist if other ED or hospital-wide improvements were made during the study period, although we are unaware of any major changes in patient care. The study was conducted by providers in the subject ED, which could have introduced bias.^13^ Some resident physicians served as data abstractors and were not blinded to study objectives. In addition, there is a trend toward more outpatient evaluation for coronary artery disease in general, which influenced our results in unclear ways. Risk factors such as obesity and smoking are tremendously under-reported in our EHR but likely consistent between cohorts.

The HEART score was derived and validated for chest pain patients. The history portion of the score is designed for chest pain patients and as expected was lower in the atypical group. This may bias the score against patients with only atypical symptoms, as it is more difficult to get a higher score for history in this group. Future studies need to have better follow up and determine the MACE rates for patients in this category.

We used the ordering of troponin and an ECG as our inclusion criteria. There are other reasons for ordering these tests together (eg, determining the physiologic burden of pulmonary embolism) but underlying cardiac disease is the primary reason for ordering these tests in the majority of these cases, even when the primary diagnosis being considered is arrhythmia, stroke, or another non-cardiac cause.^16^ Inclusion of patients where ECG and troponin were ordered when there was no concern for ACS is possible but numbers are likely low and equal between cohorts.

There was a difference in the overall health of the pre- and post-protocol populations with the pre-protocol population tending to be older with more cardiac risk factors. This was accounted for by using logistic regression to account for HEART score category (low, medium, high). This effectively controls for differences in the components of the HEART score such as age and risk factors. Results after logistic regression were slightly dampened but remained statistically and clinically significant. It is unclear as to why the populations differed. It is possible that the threshold for ordering troponin and ECGs has decreased in recent years or that our population has developed a lower threshold for presenting to the ED with mild symptoms. The decreased severity of risk factors was consistent with previous studies.^8^ It is well known that fewer patients are smoking over time, and recent publications also note a recent decrease in chest pain patients with hyperlipidemia and diabetes.^8^

Lack of follow-up occurred at a rate of 8%. This is unlikely to have changed our results substantially, particularly because rate of follow-up was similar between the two groups. The loss to follow-up hindered our ability to draw conclusions about MACE. However, external validation has previously demonstrated the safety of a HEART based protocol.^17^,^18^

### Areas for Future Research

A rule for assisting with disposition of patients with atypical symptoms of ACS is desirable. The HEART score is a good starting point. Based on our study, minor modifications to the history portion of the HEART score may be all that is required to make it more applicable to patients with only atypical symptoms. Such modification may require new derivation and validation studies. We would also encourage current and future researchers to include data on atypical patients when publishing on HEART and other cardiac risk stratification tools.

## CONCLUSIONS

After adoption of a HEART score-based protocol, discharges from the ED increased with a corresponding decrease in admissions for cardiac evaluations as well as cost. These effects were similar in patients presenting without chest pain but with presentations concerning for acute coronary syndrome.

## Figures and Tables

**Table 1:** Baseline characteristics pre- and post-adoption of HEART-based protocol.

Characteristic	Pre-implementation	Post-implementation	*p*-value
Patients			
All	844	861	NA
Typical/atypical	434/410 (51%)/(49%)	482/379 (56%)/(44%)	0.07
Gender			
All	Male: 442 (52%) Female: 402 (48%)	Male: 412 (48%) Female: 449 (52%)	0.07
Typical/atypical	Male: 218/224 (50%/55%) Female: 216/186 (50%/45%)	Male: 230/182 (48%/48%) Female: 252/197 (52%/52%)	0.5/0.07
History			
All			
0	522 (62%)	519 (60%)	0.5
1	302 (36%)	320 (37%)	
2	20 (2%)	22 (3%)	
Typical/atypical			
0	169/353 (39%/86%)	186/333 (39%/88%)	0.9/0.2
1	245/57 (56%/14%)	276/44 (57%/12%)	
2	20/0 (5%/0%)	20/2 (4%/0%)	
ECG			
All			
0	658 (78%)	636 (74%)	0.02
1	183 (22%)	225 (26%)	
2	3 (0%)	0 (0%)	
Typical/atypical			
0	356/302 (82%/74%)	369/267 (77%/70%)	0.06/0.2
1	77/106 (18%/26%)	113/112 (23%/30%)	
2	1/2 (0%/0%)	0/0 (0%/0%)	
Age			
All			
0 (<45)	183 (21%)	240 (28%)	0.01
1 (45–64)	460 (55%)	443 (51%)	
2 (≥65)	201 (24%)	178 (21%)	
Typical/atypical			
0 (<45)	136/47 (31%/11%)	179/61 (37%/16%)	0.2/0.2
1 (45–64)	237/223 (55%/54%)	245/198 (51%/52%)	
2 (≥65)	61/140 (14%/34%)	58/120 (12%/32%)	
Risk Factors			
All			
0	201 (24%)	257 (30%)	0.009
1	390 (46%)	387 (45%)	
2	253 (30%)	217 (25%)	
Typical/atypical			
0	113/88 (26%/21%)	175/82 (36%/22%)	0.004/0.3
1	201/189 (46%/46%)	195/192 (40%/51%)	
2	120/133 (28%/32%)	112/105 (23%/28%)	
Troponin category			
All			
0	757 (90%)	808 (94%)	0.007
1	64 (7%)	41 (5%)	
2	23 (3%)	12 (1%)	
Typical/atypical			
0	404/353 (93%/86%)	461/347 (96%/91%)	0.01/0.05
1	23/41 (5%/10%)	19/22 (4%/6%)	
2	7/16 (2%/4%)	2/10 (0/3%)	
HEART score			
All			
0–3 (Low risk)	577 (68%)	632 (73%)	0.06
4–6 (Medium risk)	256 (30%)	222 (26%)	
≥7 (High risk)	11 (1%)	7 (1%)	
Typical/atypical			
0–3 (Low risk)	300/277 (69%/68%)	364/268 (75%/71%)	0.10/0.5
4–6 (Medium risk)	129/127 (30%/31%)	114/108 (24%/28%)	
≥7 (High risk)	5/6 (1%/1%)	4/3 (1%/1%)	

For characteristics that are part of the HEART score, the HEART score category is included. All differences were analyzed using chi-square testing. Each characteristic is also shown according to whether they presented with typical or atypical symptoms.

*ECG*, electrocardiogram.

**Table 2:** Disposition for patients pre- and post-adoption of HEART-based protocol.

Disposition	Pre-cohort	Post-cohort	Percent change absolute	OR -*adj
Discharged				
All	428 (50.7%)	530 (61.6%)	10.8	1.56,*1.49 (P<0.001)
Typical	244 (56.2%)	326 (67.6%)	11.4	1.63,*1.57 (P<0.001)
Atypical	184 (44.9%)	204 (53.8%)	8.9	1.43, *1.38 (P = 0.01)
Admit cardiac				
All	208 (24.6%)	112 (13.0%)	−11.6	0.46, *0.47 (P<0.001)
Typical	130 (30.0%)	79 (16.4%)	−13.6	0.46, *0.44(P<0.001)
Atypical	78 (19.0%)	33 (8.7%)	−10.3	0.41, *0.43(P<0.001)
Admit to other service				
All	161 (19.1%)	177 (20.6%)	1.5	1.10, *1.15 (P = 0.5)
Typical	25 (5.76%)	41 (8.5%)	2.7	1.52, *1.51 (P = 0.1)
Atypical	136 (33.2%)	136 (35.9%)	2.7	1.13, *1.18 (P = 0.5)
ED observation				
All	35 (4.1%)	35 (4.1%)	−0.1	0.98,*0.98 (P = 1.0)
Typical	33 (7.6%)	33 (6.8%)	−0.8	0.89,*0.90 (P = 0.7)
Atypical	2 (0.5%)	2 (0.5%)	0	1.08,*1.13 (P = 1.0)
Transfer				
All	12 (1.4%)	7 (0.8%)	−0.6	0.57,*0.57 (P = 0.3)
Typical	2 (0.4%)	3 (0.6%)	0.2	1.35,*1.40 (P = 1)
Atypical	10 (2.4%)	4 (1.1%)	−1.4	0.43,*0.42 (P = 0.2)

Admission to other service was an admission which was not to the cardiology service (almost always internal medicine). Odds ratios calculated from raw data and (*) corrected for difference in baseline HEART scores for the pre-and post-protocols using logistic regression. Statistical significance evaluated with Fisher’s exact test.

*HEART*, history, electrocardiogram, age, risk factors, troponin; *ED*, emergency department; *OR adj*; odds ratio adjusted.

**Table 3:** Length of stay pre- and post-adoption of HEART-based protocol.

Length of stay	Pre-Implementation	Post-implementation	Difference	*p*-value
Discharges (n)	428	530		
Total ED time (minutes)				
All	274 ± 5.5	261 ± 4.4	13	0.07
Typical	273 ± 7.9	259 ± 5.8	14	0.1
Atypical	275 ± 7.5	265 ± 6.7	10	0.3
ED room to disposition				
All	248 ± 5.3	231 ± 4.3	17	0.01
Typical	248 ± 7.6	229 ± 5.8	19	0.04
Atypical	248 ± 7.1	235 ± 6.5	13	0.2
Admits (n)	404	324		
Inpatient days				
All	2.62 ± 0.15	2.17 ± 0.13	0.45	0.02
Typical	1.85 ± 0.16	1.54 ± 0.12	0.31	0.1
Atypical	3.30 ± 0.23	2.73 ± 0.22	0.51	0.07

Length of stay for discharged patients is evaluated in two ways. Total ED time is time from check-in until discharge. ED room to disposition excludes waiting room time from length of stay. Inpatient length of stay is in days and includes patients dispositioned to ED observation, cardiac admission and admissions to other services. Data is included as means ± SEM. P-values were calculated using two-sided Student’s t test.

*HEART*, history, electrocardiogram, age, risk factors, troponin; *ED*, emergency department.

**Table 4:** Cardiac testing. Stress testing and cardiac catheterizations performed pre- and post-adoption of HEART-based protocol.

Testing	Pre-cohort	Post-cohort	OR -*adj	*p*-value
Stress testing				
Performed				
All	138 (16%)	72 (8%)	0.47,*0.49	<0.001
Typical	115 (26%)	64 (13%)	0.43,*.0.44	<0.001
Atypical	23 (6%)	8 (2%)	0.36,*0.38	0.02
Abnormal result				
All	20 (14%)	11 (15%)	0.99,*0.95	1.0
Typical	13 (11%)	10 (16%)	1.34,*1.30	0.6
Atypical	7 (30%)	1 (13%)	0.34,*0.29	0.6
Catheterizations				
Performed				
All	32 (4%)	26 (3%)	0.79,*0.90	0.4
Typical	24 (6%)	17 (4%)	0.63,*0.67	0.2
Atypical	8 (2%)	9 (2%)	1.23,*1.46	0.8
Abnormal (%)				
All	17 (53%)	20 (77%)	2.89,*1.91	0.1
Typical	14 (58%)	14 (82%)	3.24,*2.38	0.2
Atypical	3 (38%)	6 (67%)	3.09,*1.78	0.3

Odds ratios calculated from raw data and (*) corrected for total HEART scores for the pre and post protocols using logistic regression. Statistical significance was evaluated with Fisher’s exact test.

*HEART*, history, electrocardiogram, age, risk factors, troponin.

**Table 5:** Healthcare cost pre and post adoption of HEART-based protocol.

Event	Cost per	Pre (annual)	Post (annual)	Annual change	Savings (cost)
Cardiac admit	$22,257	208 (696)	112 (417)	−279	$6,217,958
General admit	$9,111	161 (539)	177 (659)	+120	($1,094,288)
Cardiology visit	$297	428 (1433)	530 (1974)	+541	($586,272)
Stress test	$277	138 (462)	72 (268)	−194	$53,723
Total					$ 4,591,121

Estimates of healthcare costs based on changes on admission rate, stress testing, catheterizations, etc.
